# Effect of a One‐Week Lubricant Pretreatment on Post‐LASIK Dry Eye: Single‐Center Randomized, Single‐Masked Study With 1‐Month Follow‐Up

**DOI:** 10.1155/joph/6643075

**Published:** 2026-06-22

**Authors:** Rafael Cañones-Zafra, German Allendes-Urquiza, Cristina Muñiz, Miguel A. Teus

**Affiliations:** ^1^ Ophthalmology Department, Clinica Novovisión, Madrid, Spain; ^2^ Ophthalmology Department, Hospital Universitario Principe de Asturias, Alcalá de Henares, Spain; ^3^ Ophthalmology Department, Universidad de Alcalá, Alcalá de Henares, Spain, uah.es

**Keywords:** dry eye, Femto-LASIK, lubricants

## Abstract

**Purpose:**

Our purpose is to report the results of a prospective study on ocular discomfort and objective tear film parameters (osmolarity and NIKBUT), in myopic patients, and observe the possible differences at 1 month between randomized groups in the use of topical lubricants 1 week prior to surgery or just after surgery.

**Methods:**

This is a prospective, single masked, randomized, comparative study. Refractive error and UDVA, BCVA, tear osmolarity, OSDI test, and keratograph analysis of the tear film were performed per patient by a masked examiner before and 1 day, 1 week, and 1 month after FS‐LASIK performed to correct myopia in both pre and post groups. A total of 22 patients per group were included.

**Results:**

Mean age was 32.27 ± 5.4 and 32.85 ± 5.3 years, and mean preop sphere was −3.17 ± 2.3 and 2.75 ± 1.3 D in pre and post groups, respectively (*p* > 0.05). In addition, no statistically significant difference was found in preoperative OSDI, tear osmolarity, or NIKBUT between both groups. In the whole cohort, the OSDI worsened significantly from a preop value of 6.94 ± 7.15 to a value of 15.66 ± 12.55 at 1‐month postop (*p* < 0.0001).

We did not find any statistically significant differences in the OSDI score, NIKBUT, or osmolarity at the 1‐month postop between the pre and post groups.

**Conclusion:**

Femto‐LASIK increased OSDI at 1 month. One‐week pretreatment with hyaluronate–HP‐guar did not improve symptoms, osmolarity, or NIKBUT compared with postoperative use only. More trials with longer follow‐up are needed.

**Trial Registration:** Spanish Agency of Medicines and Medical Devices (AEMPS): Code 25‐0055

## 1. Introduction

Tear stability has clinical relevance as it may affect visual acuity and quality of vision and is related with discomfort after refractive surgery. Dry eye symptoms continue to be one of the main causes of dissatisfaction after Femto‐LASIK surgery [1–3], affecting not only the expected visual outcomes but also the quality of life [4, 5], with an expected worsening in the OSDI values as indicator of symptoms of dry eye [6, 7].

Artificial tears are commonly used postoperatively to increase the viscosity and stability of the tear film. This helps alleviate discomfort symptoms and seem to reduce inflammation [2]. Several studies have evaluated the effects of different artificial tears on postoperative symptoms and signs following refractive surgery [2, 5, 8].

The combination of hyaluronic acid and HP‐guar has been shown to be superior to other artificial tears in managing postoperative symptoms and promoting re‐epithelialization after PRK [9]. The use of the combination of hyaluronic acid and HP‐guar continues to be studied for the management of ocular surface symptoms associated with ocular surgery, and there are some studies recommending the preoperative use of this combination for cataract surgery, as this seems to improve the postoperative dry eye symptoms [10]. There is no evidence in the literature suggesting that in laser refractive surgery, the use of this formulation before surgery can have beneficial effects on the postoperative dry eye symptoms.

The purpose of this study is to evaluate the effects of pretreatment with artificial tears containing the combination of hyaluronic acid and HP‐guar on postoperative dry eye and objective tear film parameters in myopic patients scheduled for bilateral Femto‐LASIK surgery, randomized to use Systane HYDRATION MDPF either starting 1 week before surgery (pretreatment group) or starting after surgery (no pretreatment group).

## 2. Methods

This prospective, single‐masked, randomized, comparative study was conducted at the Clínica Novovisión Madrid, Spain. The study followed the principles established in the Declaration of Helsinki (1964), in the Council of European Convention on Human Rights and Biomedicine (1997), and the regulations on biomedical research and personal data protection of the Regional Ethics Committee (CEIm‐R).

Written informed consent was obtained from all the patients for both participation in the study and the publication of the results, with an updated data protection policy appendix.

Patients 18 years of age or older with myopia with or without astigmatism and candidates for FS‐LASIK were selected. They had to present an OSDI greater or equal to 5 points and less than 12 points, have healthy corneas with no associated ocular or systemic pathology, and not be taking medications that could have ocular effects.

The study included 44 patients undergoing myopic Femto‐LASIK. All patients were treated with Systane HYDRATION MDPF (sodium hyaluronate 0.15%, polyethylene glycol 400 0.4%, propylene glycol 0.3%, and hydroxypropyl guar 0.175%, Alcon Laboratories, Fort Worth, TX, USA) lubricating eye drops. Twenty‐two patients were treated according to the standard postoperative protocol, receiving only lubrication drops after surgery 4 times a day (standard control group). Another 22 were pretreated 1 week before surgery with the same lubricant (study or pretreatment group) and continued after surgery with the same lubrication regimen as the control group. Patients were randomized to each treatment group using a computer generated list. The randomization was done when the surgery was scheduled by the patient, so at that time, the patient was told to use the Systane HYDRATION MDPF either before or just after surgery by an independent assistant.

All surgeries were performed by the same surgeon (MAT), in the same center and with the same protocols (superior flap location and with the same postoperative protocol. Any need for modification or increases in medication was considered as exclusion criteria. In both groups, the ocular surface was evaluated by the same masked examiner. All the data were analyzed by a masked investigator.

Preoperative and postoperative evaluations included the uncorrected and corrected distance visual acuities (UDVA and CDVA, in decimal notation), the ocular surface disease index (OSDI) questionnaire, tear osmolarity (Tear lab, Escondido, CA, USA), and noninvasive Keratograph break up time (NIKBUT) (Keratograph, Oculus, Wetzlar, Germany). The tear osmolarity and NIKBUT exams were performed in both eyes of each subject and then the values obtained in both eyes at each examination were averaged. The following measurements were performed:

• OSDI: before surgery, 1 day, 1 week, and 1 month after surgery.
• Osmolarity: before surgery, 1 day, and 1 month after surgery.
• NIKBUT: before surgery, 1 week, and 1 month after surgery.

### 2.1. Sample Size

A sample size of 22 patients per group was considered appropriate, in a 2‐arm parallel design, CI = 95%, and *p* = 80%, with expected differences of 5.61 in OSDI and with a standard deviation (SD) of 6.46. The expected differences in OSDI and its SD were extracted from the study by Favuzza et al. [10].

### 2.2. Statistical Analysis

The statistical analysis was done using StatPlus (AnalystSoft Inc. Alexandria, Virginia, USA). The normality of the data distribution was checked using the Kolmogorov–Smirnov test. Continuous variables are shown as the mean followed by the SD, and the VA values are shown in decimal scale notation. The Student’s *t*‐test and the ANOVA tests were used for comparisons when appropriate.

## 3. Results

The mean age of the patients in the pretreatment and no pretreatment groups was 32.27 ± 6.4 and 32.13 ± 5.3 years, respectively (*p* = 0.9). Mean preoperative sphere was −3.14 ± 2.3 and −2.63 ± 1.3 D in the pretreatment and no pretreatment groups, respectively (*p* = 0.2). There were no significant differences between the two groups in terms of gender, preoperative refractive error magnitude, or preoperative ocular surface parameters, as can be seen in Table 1.

**TABLE 1:** Demographics and preoperative examination results in groups’ study.

	Pretreatment group	No pretreatment group	*p*
Age	32.27 ± 6.4	32.13 ± 5.6	0.9
Sphere	−3.14 ± 2.3	−2.63 ± 1.3	0.2
Cylinder	−0.89 ± 0.9	−0.70 ± 0.5	0.4
BCVA	1.16 ± 0.07	1.18 ± 0.1	0.5
NIKBUT ini (sec)	11.08 ± 6.8 (9.01–13.14)	11.82 ± 8.5 (9.23–14.42)	0.6
NIKBUT med (sec)	12.05 ± 6.5 (10.06–14.04)	13.16 ± 7.8 (10.76–15.56)	0.4
OSMO (mOsm/L)	301.00 ± 12.89 (293.52–302.83)	299.63 ± 10.03 (296.75–302.92)	0.2
OSDI	6.34 ± 6.08 (4.49–8.19)	7.94 ± 7.89 (5.54–10.35)	0.2

*Note:* NIKBUT and Osmolarity and OSDI presented with their 95% CIs (lower control limit and upper control limit). NIKBUT (seg): noninvasive Keratograph tear breakup time.

Abbreviations: BCVA, best‐corrected visual acuity; OSDI, ocular surface disease index.

Postoperatively, both groups showed no difference in visual acuity at any follow‐up point. Uncorrected visual acuity at 1‐day postop was 1.05 ± 0.18 in the pretreatment group and 1.00 ± 0.2 in no pretreatment group (*p* = 0.3). At 1 month after surgery, it was 1.07 ± 0.15 in the pretreatment group and 1.09 ± 0.17 in no pretreatment group (*p* = 0.6). No relevant complication or adverse events were noted throughout the study.

Similarly, there were no differences in tear osmolarity between the groups on the day after surgery or 1 month after Femto‐LASIK (*p* = 0.2). In addition, no significant differences in dry eye symptoms, as assessed by OSDI, were observed postoperatively between the groups. The pretreatment group showed an OSDI the day after surgery of 13.98 ± 13.04 and no pretreatment group had an OSDI of 18.59 ± 19.09 (*p* = 0.1). One month after surgery, OSDI scores were 16.05 ± 12.81 and 14.61 ± 12.06 in the pretreatment and no pretreatment groups, respectively (*p* = 0.5). There was a significant increase in OSDI scores after surgery across the entire cohort (Table 2).

**TABLE 2:** Ocular surface disease index (OSDI) scores for the entire cohort presented with their 95% CIs (lower control limit and upper control limit).

	Preop	Day 1	Day 7	1 month
OSDI score	7.14 ± 7.05 (5.65–8.64)	16.28 ± 16.41 (12.80–19.76)	11.22 ± 19.19 (16.81–21.57)	12.39 ± 15.33 (12.70–17.95)

Tear stability, measured by NIKBUT, also showed no significant differences at 1 week and 1 month postoperatively. The parameters assessed are detailed in Table 3.

**TABLE 3:** Postoperative visual outcomes.

		1 day postop	*p*	1 week postop	*p*	1 month postop	*p*
UCVA	Pretreatment	1.05 ± 0.18	0.3	1.04 ± 0.18	0.3	1.07 ± 0.15	0.6
	No pretreatment	1.00 ± 0.2		1.01 ± 0.16		1.09 ± 0.17	

OSMO (mOsm/L)	Pretreatment	303.09 ± 11.71 (293.30–305.58)	0.2	NA		300.29 ± 11.72 (296.72–303.86)	0.2
	No pretreatment	299.44 ± 19.94 (299.52–306.55)				297.15 ± 12.33 (293.40–300.91)	

OSDI	Pretreatment	13.98 ± 13.04 (10.01–17.64)	0.1	19.38 ± 10.04 (15.72–23.04)	0.8	16.05 ± 12.81 (12.15–19.84)	0.5
	No pretreatment	18.59 ± 19.09 (12.78–24.39)		19.00 ± 10.49 (15.81–22.19)		14.61 ± 12.06 (10.94–18.28)	

NIKBUT ini (sec)	Pretreatment	NA		9.81 ± 8.48 (7.68–11.95)	0.5	10.63 ± 7.6 (8.31–12.96)	0.6
	No pretreatment			10.89 ± 7.01 (8.31–13.41)		11.28 ± 7.6 (8.90–13.66)	

NIKBUT med (sec)	Pretreatment	NA		11.40 ± 6.5 (9.43–13.38)	0.3	13.14 ± 7.5 (10.35–14.76)	0.7
	No pretreatment			12.85 ± 7.5 (10.56–15.15)		12.5 ± 7.2 (10.84–15.43)	

*Note:* Osmolarity, OSDI and NIKBUT, presented with their 95% CIs (lower control limit and upper control limit). UCVA: uncorrected distance visual acuity, OSMO: osmolarity, NIKBUT med (Seg): noninvasive Keratograph tear breakup time.

Abbreviation: OSDI, ocular surface disease index.

Comparing preoperative and postoperative values, the surgery did not induce significant changes in either tear osmolarity or stability, with no significant differences observed between the groups although the postoperative OSDI score was significantly worse at any postop visit compared with the preoperative values (Table 4).

**TABLE 4:** Comparison of the osmolarity, NIKBUT, and OSDI scores in both groups before and after surgery.

		**Before surgery**	**1 day**	**1 month**	**p**

Osmo (mOsm/L): Pretreatment		301.00 ± 12.89	303.09 ± 11.71	300.29 ± 11.72	0.5
Osmo (mOsm/L): No pretreatment		299.63 ± 10.03	299.44 ± 19.94	297.15 ± 12.33	0.6

		**Before surgery**	**7 days**	**1 month**	**p**

NIKBUT med (secs): Pretreatment		12.05 ± 6.5	11.40 ± 6.5	13.14 ± 7.5	0.8
NIKBUT med (secs): No pretreatment		13.16 ± 7.8	12.85 ± 7.5	12.5 ± 7.2	0.7

	**Before surgery**	**1 day**	**7 days**	**1 month**	**p**

OSDI: Pretreatment	6.34 ± 6.08	13.98 ± 13.04	19.38 ± 10.04	16.05 ± 12.81	0.001
OSDI: No pretreatment	7.94 ± 7.89	18.59 ± 19.09	19.00 ± 10.49	14.61 ± 12.06	0.001

*Note:* Femto‐LASIK surgery. Osmo: osmolarity, NIKBUT med (Seg): noninvasive Keratograph tear breakup time.

Abbreviation: OSDI, ocular surface disease index.

## 4. Discussion

Dry eye is one of the most frequent complications after Femto‐LASIK. There are several studies that recommend treatment with artificial tears in the postoperative period, as they reduce symptoms and inflammation [2–4]; however, there is a lack of research on the effects of pretreatment with artificial tears in Femto‐LASIK surgery performed to correct myopia.

Our study demonstrated that pretreatment with artificial tears 1 week before surgery did not improve dry eye symptoms or signs at 1 month in this cohort with mild baseline symptoms.

There was a significant increase in OSDI score postsurgery across the entire cohort, as expected in the early postop of LASIK. This fact has been reported in previous studies using artificial tears containing HA after Femto‐LASIK [11]. However, it must be stressed that the OSDI increase at one month is close to 10 points, which has been considered the minimal clinically important difference, thus supporting that LASIK induced dry eye symptoms are usually mild.

Tear stability, evaluated by the NIKBUT, showed no significant difference between the two groups, and in addition, this parameter did not worsen after surgery. The same results were obtained in tear osmolarity. These findings agree well with the ones obtained by Tsai et al. and Sauvageot [11, 12], who did not observe significant differences in neither the BUT or tear osmolarity between the preoperative values and those measured during the early postoperative period. This discrepancy between the objective parameters and symptoms of dry eye agree well with the “phantom cornea” hypothesis, which suggests that it is the process of regeneration of severed corneal nerves the main cause of LASIK induced dry eye symptoms [13].

Our study has some limitations.

The small sample of patients in both arms of the study in a single center, and that the patient adherence to treatment was not checked objectively, could limit significant differences between both groups studied, and the follow‐up time of only 1 month may affect the results.

In addition, the cohort had none or mild baseline dry eye symptoms, what can limit detectable improvement. The choice to include only patients with low or normal OSDI values could also introduce a floor effect, potentially masking the benefits of pretreatment in symptomatic individuals. Nevertheless, we believe that our patient population is quite similar to the usual LASIK candidates because preoperative moderate and severe dry eye is considered to be a contraindication for this procedure.

Therefore, further studies should be conducted to overcome this and provide additional data, for example, on the effects of different types of pretreatments [2, 14], different surgical techniques, and different degrees of refractive error or to compare the effects of the lubricant combination used, with other available options for the management of dry eye [15].

However, to the best of our knowledge, our study is the first to evaluate the effect of pretreatment with topical lubricants in femtosecond LASIK surgery performed to correct myopia.

## 5. Conclusion

According to our results, we may conclude that pretreatment with artificial tears is not necessary to improve discomfort or tear stability after FS‐LASIK surgery is performed to correct myopia, only an adequate postoperative lubricating regime seems to be necessary to relieve the ocular discomfort. This is likely because younger populations undergoing laser vision correction generally have a healthier ocular surface as compared with older patients candidates for cataract surgery.

The absence of deterioration in both the tear osmolarity and stability postoperatively, together with the significant worsening in the OSDI score, supports the hypothesis that the mismatch between symptoms and signs may suggest a neuropathic component. It is clear that more studies with longer follow‐up and including an evaluation of the corneal sensitivity are needed.

## Funding

This study was partially supported by an investigator‐initiated study grant from Alcon Healthcare (IIT#67952279).

## Disclosure

This study was presented at the 42nd Congress of ESCRS, Fira de Barcelona, Spain, 6–10 September 2024.

## Conflicts of Interest

The authors declare no conflicts of interest.

## Data Availability

The data that support the findings of this study are available on request from the corresponding author. The data are not publicly available due to privacy or ethical restrictions.
